# Performance of an Artificial Intelligence Support System on Screening Mammography Cases Proceeding to Stereotactic Biopsy

**DOI:** 10.3390/cancers17233878

**Published:** 2025-12-04

**Authors:** Anandita Mathur, Colleen McNally, Arielle Sasson, Nicholas Thoreson, Sadaf Sahraian, David S. Mendelson, Laurie R. Margolies

**Affiliations:** Department of Diagnostic, Molecular and Interventional Radiology, Icahn School of Medicine at Mount Sinai (ISMMS), 1470 Madison Ave., New York, NY 10029, USA; anandita.mathur2@mountsinai.org (A.M.);

**Keywords:** screening mammography, artificial intelligence, breast cancer, stereotactic biopsy

## Abstract

**Background/Objective:** The objective was to evaluate the standalone performance of an AI system, Transpara 1.7.1 (ScreenPoint Medical), in screening mammography cases proceeding to stereotactic biopsy using histopathological results as ground truth. **Methods:** This retrospective study included 202 asymptomatic female patients (mean age: 57.8 years) who underwent stereotactic biopsy at a multicenter academic institution between October 2022 and September 2023 with a preceding screening mammogram within 14 months. Transpara AI risk scores were compared to pathology results (benign versus malignant). Performance metrics for AI including sensitivity, specificity, positive predictive value (PPV), negative predictive value (NPV), and area under the receiver operating characteristic curve (AUC) were calculated. **Results:** Transpara AI classified 20 of 39 malignant findings (51%) as elevated risk compared with 50 of 211 total findings (24%). AI score was positively correlated with malignancy (r = 0.29, *p* < 0.001). Sensitivity for detecting malignancy (classifying as intermediate or elevated risk) was 94.9% (95% CI: 81.4–94.1), specificity was 24.4% (95% CI: 18.3–31.7), PPV was 22.2% (95% CI: 16.3–29.4), and NPV was 95.5% (95% CI: 83.3–99.2). Transpara had fair performance in detecting breast cancer with AUC 0.73 (95% CI: 0.63–0.82). **Conclusions:** Transpara AI is a useful screening mammography triage tool. Given its high sensitivity and high negative predictive value, AI may be used to guide radiologists in making biopsy or follow up recommendations. However, the high false-positive rate and presence of two false negatives underscore the need for radiologists to use caution and clinical expertise when interpreting AI results.

## Simple Summary

Digital breast tomosynthesis (DBT) for screening mammography is widely adopted in the United States and deep learning-based artificial intelligence (AI) algorithms have been developed to increase accuracy and efficiency in breast cancer detection. Few studies have evaluated the performance of AI decision support in real-world settings, especially on DBT exams. Transpara AI decision support was recently integrated into the clinical workflow for screening mammography at our institution and our aim was to assess its standalone performance in detecting breast cancer on DBT cases proceeding to stereotactic biopsy. This article presents performance metrics for Transpara AI along with representative cases of AI misses and false positives to provide insights into how radiologist–AI synergy can improve patient care.

## 1. Introduction

Breast cancer is the most frequently diagnosed cancer in women with an incidence of 13.1% and leads to approximately 42,780 deaths annually in the United States [1]. The combination of early detection through mammographic screening and treatment advances has led to an estimated 44% mortality decrease since 1989 [1]. Digital breast tomosynthesis (DBT), a quasi-three-dimensional imaging technique approved by the FDA in 2011, is widely implemented in the United States with over 90% of Mammography Quality Standards Act (MQSA)-certified facilities reporting units in 2024. DBT functions by taking multiple low dose X-rays from different angles along an arc and reconstructing the images to create a 3D scrollable stack, resulting in superior visualization of overlapping breast tissues. DBT has been shown to improve sensitivity and specificity while reducing recall rate compared to 2D digital mammography, as demonstrated by numerous studies and a meta-analysis [2,3,4]. However, DBT’s principal drawback is increased radiologist workload as interpretation times are about twice as long as 2D digital mammography [5].

Artificial intelligence (AI), a rapidly evolving field using computer-based algorithms to perform tasks, is transforming breast imaging with applications in diagnosis, risk-stratification, breast density assessment, image quality control, and prediction of treatment response [6,7,8,9]. Significant progress has been made in leveraging deep learning-based AI algorithms to improve the accuracy and efficiency of screening mammography. Deep learning convolutional neural networks (CNNs) are used to segment and classify images based on trained models from large datasets [10]. Several retrospective studies and the Mammography Screening with Artificial Intelligence (MASAI) randomized control trial have demonstrated that radiologists had higher accuracy for detecting cancer on 2D digital mammography with AI support than with unaided reading [11,12,13,14]. Additionally, large retrospective studies have shown that AI strategies may cut reading time in half and dramatically reduce the workload of screening mammography [15,16,17].

Prior to the rise of AI, traditional machine learning computer-aided detection (CAD) systems were implemented since 1998, which marked an unlimited number of suspicious lesions on mammograms based on pre-programmed features. However, the established benefit of CAD has been challenged as several studies have shown that CAD does not improve accuracy and increases biopsy rates [18,19]. Moreover, CAD was shown to increase the time needed to interpret each study by approximately 20% due to a high rate of false positives [20]. Deep learning-based AI algorithms have been shown to outperform CAD with significantly better sensitivity and specificity when applied to a single mammography dataset [21]. Additional advantages of AI algorithms which classify data for a specific task include the potential to identify imaging features beyond the limits of human detection and to continuously improve [7,22].

With the overall goal of improving patient care, our institution replaced CAD in 2022 with a commercially available AI system for DBT, Transpara 1.7.1. A retrospective study in an enriched cohort with 25% cancer cases comparing two DBT AI systems (Transpara and ProFound AI) against radiologists found high AI performance in breast cancer detection but lower performance compared with radiologists [23]. However, a meta-analysis by Yoon et al. including four studies suggested that standalone AI had better sensitivity and specificity compared to radiologists in interpreting DBT exams, although there were insufficient studies to assess performance [24]. There is a paucity of studies evaluating the utilization of AI for DBT interpretation in a real-world setting versus in cancer-enriched datasets [25]. A retrospective real-world study by Letter et al. found nonsignificant improvement in cancer detection rate (CDR) with AI support, while a recent retrospective real-world study of 6407 DBT exams by Nepute et al. demonstrated a significant improvement in CDR [26,27]. It is crucial to understand the strengths and limitations of AI decision support for DBT in real clinical practice to guide radiologists in making biopsy or follow-up recommendations.

The objective of this study is to evaluate how Transpara AI decision support performed in detecting breast cancer on screening DBT mammography cases proceeding to stereotactic biopsy with histopathological results as the ground truth.

## 2. Methods

### 2.1. Study Population

This single-institution retrospective study was compliant with the Health Insurance Portability and Accountability Act. The institutional review board approved the study and waived the requirement of informed consent.

This study was performed at four urban centers within a single academic health system. We searched our Mammography Information System (MIS) (Ikonopedia, Richardson, TX, USA) for all stereotactic breast biopsies for adult patients from 1 October 2022 to 30 September 2023, resulting in 1051 biopsies.

**Figure 1:**
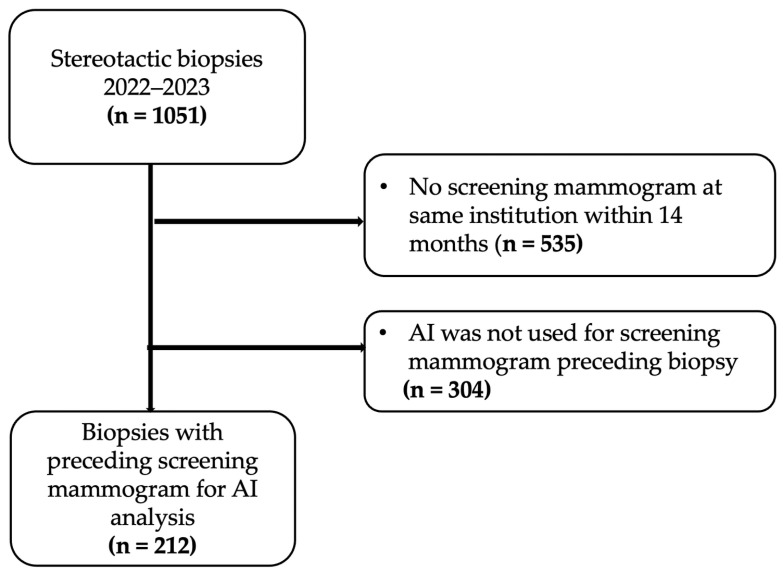
Study flow chart.

Patients were then excluded for the following reasons: no preceding screening mammogram at the same institution within 14 months of biopsy (535 biopsies) or AI was not used for preceding mammogram (304 biopsies). All patients who underwent screening mammograms were asymptomatic and biopsies performed based on recommendations from diagnostic mammograms or ultrasound were excluded as the objective was to evaluate the performance of artificial intelligence on screening mammograms. Figure 1 shows the flow of patient selection, which resulted in 212 stereotactic biopsies with preceding screening mammogram for AI analysis.

The following information was extracted from our MIS: age, self-reported race and ethnicity, breast density on screening mammogram, finding type on mammography exam (classified as calcifications, mass, asymmetry, or architectural distortion), personal history of breast cancer, and family history of breast cancer in a first-degree relative. Race and ethnicity were evaluated because of their potential association with breast cancer screening. Race and ethnicity were categorized into the following broad categories: White, Black/African American, Asian, Hispanic, and unknown. Patients with Hispanic ethnicity were categorized as Hispanic regardless of race.

### 2.2. Image Acquisition

Patients underwent screening mammography using digital breast tomography (DBT) (Hologic, Marlborough, MA, USA). All breast imaging examinations were interpreted by dedicated breast radiologists based on the BI-RADS Atlas (5th edition).

This study used Transpara 1.7.1 (ScreenPoint Medical, Nijmegen, The Netherlands), a commercially available AI designed for automated breast cancer detection in screening mammography and breast tomosynthesis processed images. Transpara 1.7.1 does not evaluate prior examinations in risk score assessment. Transpara generates an interactive decision support score between 1 and 100 (score of 100 indicates the highest suspicion of malignancy) for each lesion and an overall case score which represents the highest score of all lesions. Transpara marked a maximum of two lesions per breast (most suspicious) for this dataset and provided a score category defined by the vendor as follows: low risk (≤42), intermediate risk (43–74), and elevated risk (≥75). The exact numerical value was not available for lesions classified as low risk (≤42) and thus the low-risk lesions were considered to have a score of zero. The Transpara system was trained and validated using a proprietary multicenter mammogram database and the mammograms in this study were not used to train the algorithms. All false-negative cases were reported to ScreenPoint Medical as part of quality assurance; no other support was received from ScreenPoint for this study.

### 2.3. Ground Truth

All patients underwent stereotactic biopsy and histopathology results served as the ground truth. The histologic types of breast cancer included ductal carcinoma in situ (DCIS), invasive ductal carcinoma (IDC), and invasive lobular carcinoma (ILC). There was one case of malignant lymphoma which was excluded from the analysis. Atypical lesions with high-risk features were noted and included atypical ductal hyperplasia (ADH), atypical lobular hyperplasia (ALH), lobular carcinoma in situ (LCIS), radial scar/complex sclerosing lesion, and papilloma with ADH. Atypical lesions and all other histopathology results were considered benign. Transpara lesion-specific scores were compared to pathology results on the biopsy sample (benign versus malignant). Two study authors reviewed all stereotactic biopsy mammogram images in conjunction with the preceding screening mammogram to obtain lesion-specific scores and ensure the location of the AI markings corresponded to the biopsied lesion. If multiple lesions from a single patient underwent stereotactic biopsy, each lesion was considered independent and lesion-specific score was used for evaluating AI performance.

### 2.4. Statistical Analysis

Data was summarized descriptively, and all analyses were performed using R (R Foundation for Statistical Computing, Vienna, Austria, version 4.4.1). Statistical significance was set at *p* < 0.05.

A 2 × 2 contingency table was created by calculating true-positive, false-positive, true-negative, and false-negative findings from the reported data. For the null hypothesis that there is no association between AI risk classification (low vs. intermediate/elevated risk) and biopsy outcome, *p* was less than 0.001 suggesting significant association. We calculated sensitivity, specificity, positive predictive value (PPV), and negative predictive value (NPV).

The diagnostic performance of AI for detecting malignant lesions was assessed using receiver operating characteristic (ROC) analysis. AI performance was also stratified by lesion type (mass, calcification, asymmetry, and architectural distortion). Correlation between AI risk score and malignancy was estimated using the Pearson correlation coefficient.

## 3. Results

### 3.1. Patient Characteristics

The final study sample comprised 202 patients. Of these patients, eight patients underwent biopsy of two lesions (same breast or both breasts) and one patient underwent biopsy of three lesions (both breasts).

Table 1 summarizes patient characteristics. All patients were female. The mean age for the patients was 57.7 years (range, 35–89 years). A total of 24 out of 202 (12%) patients had a prior breast cancer diagnosis. A total of 50 out of 202 (25%) patients had a family history of breast cancer. Breast density was as follows: 5% fatty, 43% scattered, 46% heterogenous, and 6% extremely dense. Patient’s self-identified race and ethnicity classifications were as follows: 44% White, 17% Black/African American, 13% Asian, 20% Hispanic, and 6% unknown.

### 3.2. Lesion Characteristics

The distribution of stereotactic biopsy lesions was as follows: 10 (5%) masses, 162 (76%) calcifications, 15 (7%) asymmetries, and 25 (12%) architectural distortions. Calcifications are typically occult on ultrasound (unless there is an associated mass) whereas masses, asymmetries, and architectural distortion are more likely to have an ultrasound correlate.

All lesions in this study were interpreted as high risk by the radiologist on screening mammogram (BIRADS 0, 3, 4, 5) as further recommendation was to undergo stereotactic biopsy. There were no low-risk lesions (BIRADS 1, 2) as these patients would have returned to screening rather than proceeding to stereotactic biopsy. The eight patients who received a BIRADS score of 3, 4, or 5 all presented for screening and the radiologist converted the screening mammogram to a diagnostic mammogram due to an abnormality.

Stereotactic biopsy results showed 136 benign, 36 high-risk lesions, and 40 malignant lesions. Table 2 summarizes the lesion characteristics including sub-types of the malignant findings.

### 3.3. AI Performance

**Figure 2:**
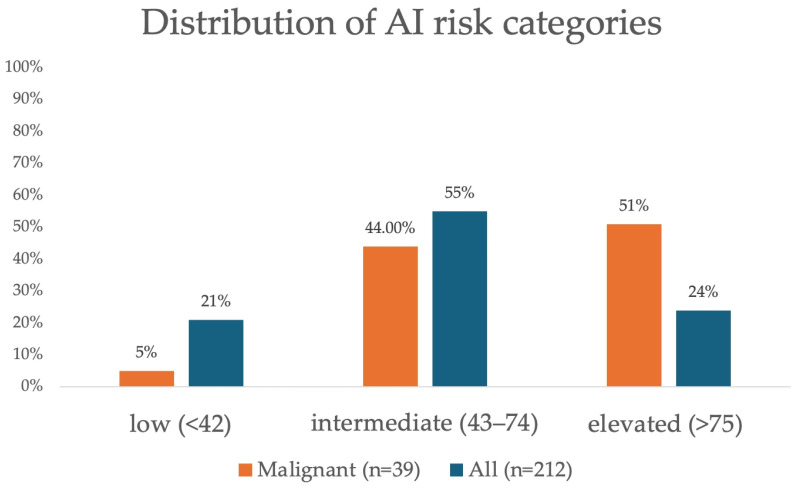
Distribution of artificial intelligence (AI) risk categories for all findings (n = 212) and malignant findings (n = 39).

Of the 212 total lesions, AI classified 45 (21%) lesions as low risk, 117 (55%) lesions as intermediate risk, and 50 (24%) lesions as elevated risk. Of the 39 malignant lesions, AI classified 2 (5%) as low risk, 17 (44%) as intermediate risk, and 20 (51%) as elevated risk. Figure 2 shows the distribution of AI score for all lesions and for malignant lesions.

Correlation with histopathological ground truth revealed 37 AI true positives, 130 AI false positives, 2 AI false negatives, and 42 AI true negatives. AI sensitivity for detecting malignancy (classifying as intermediate or elevated risk) was 94.9% (95% CI: 81.4–94.1) and specificity was 24.4% (95% CI: 18.3–31.7). PPV was 22.2% (95% CI: 16.3–29.4) for intermediate or elevated risk findings and low risk had a NPV of 95.5% (95% CI: 83.3–99.2) (Table 3). We did not find a statistically significant difference for sensitivity or specificity when stratifying patients by age, race and ethnicity, or breast density (*p* > 0.05).

**Figure 3:**
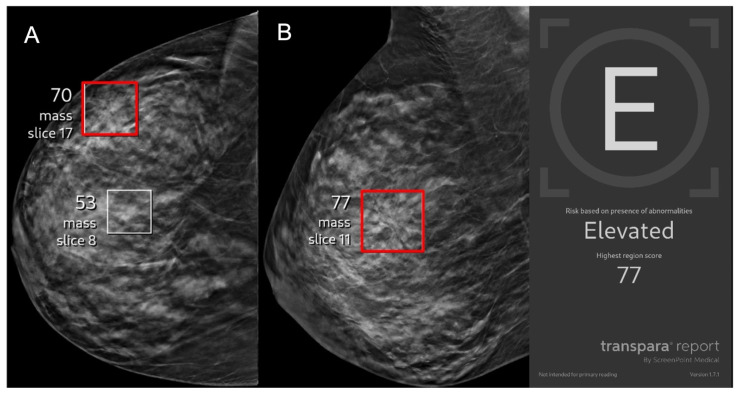
A 43-year-old asymptomatic female patient presents for screening. (**A**) Right craniocaudal and (**B**) mediolateral oblique views demonstrate an architectural distortion (red boxes) that was flagged by AI with a lesion-specific score of 77 corresponding to elevated risk. The case was recalled by the radiologist and biopsy revealed invasive ductal carcinoma, representing an AI true positive.

**Figure 4:**
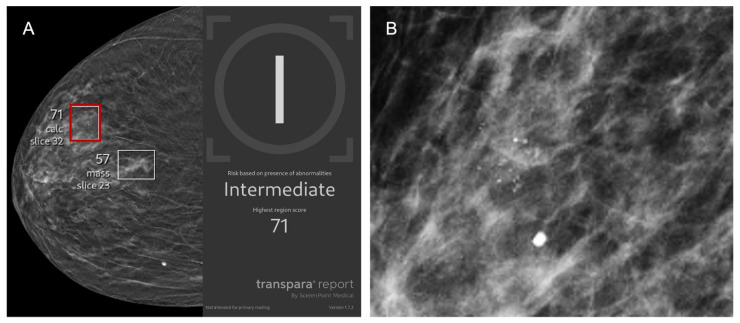
A 77-year-old asymptomatic female patient presents for screening. (**A**) Right craniocaudal and (**B**) magnification view demonstrate calcifications (red box) that were flagged by AI and assigned a lesion-specific score of 71 corresponding to intermediate risk. The case was recalled by the radiologist and biopsy revealed benign breast calcifications, representing an AI false positive.

**Figure 5:**
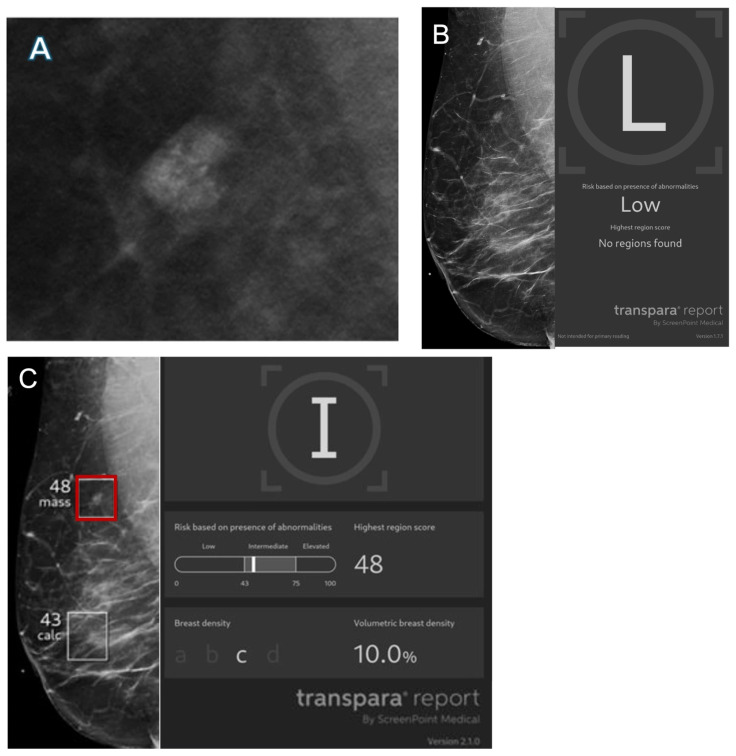
Images from a 56-year-old female patient. (**A**) Mass with calcifications on diagnostic mammogram recalled by the radiologist and found to represent ductal carcinoma in situ on biopsy. (**B**) Right mediolateral view with no finding flagged by Transpara 1.7.1 and classified as low risk, representing an AI false negative. Case was sent to Screenpoint Medical as part of quality assurance. (**C**) Right mediolateral view with lesion (red box) flagged by newer version Transpara 2.1.0 and classified as intermediate risk with a lesion-specific score of 48.

**Figure 6:**
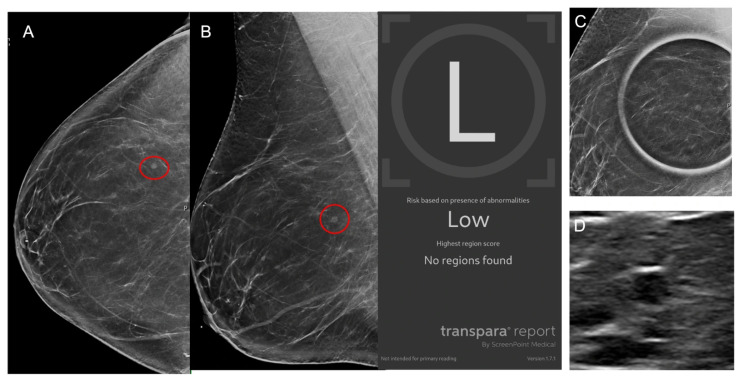
Images from a 42-year-old female patient. (**A**) Right craniocaudal and (**B**) mediolateral views demonstrate a mass (red circles) which was recalled by the radiologist although no finding was flagged by AI which classified the case as low risk. (**C**) Mass persists on spot compression view and (**D**) ultrasound demonstrates an oval hypoechoic mass. Biopsy revealed benign breast tissue with a microcyst, representing an AI true negative.

Representative images of AI true-positive, false-positive, false-negative, and true-negative cases are shown in Figure 3, Figure 4, Figure 5 and Figure 6.

AI score was positively correlated with rate of malignancy (r = 0.29, *p* < 0.001). Higher AI score categories corresponded to increased PPV (Table 4). Elevated risk (≥75) had a PPV of 40%.

**Figure 7:**
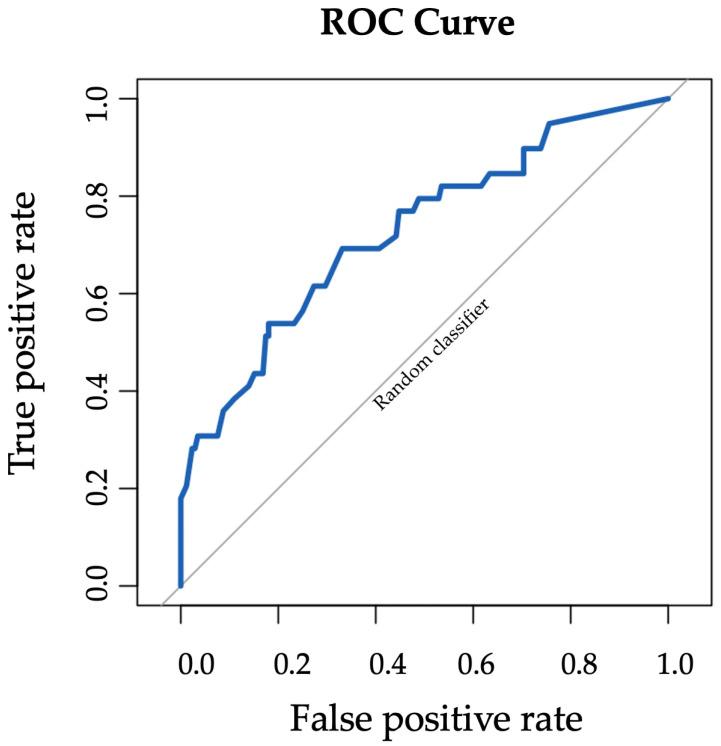
ROC curve for Transpara AI performance in detecting breast cancer with AUC 0.73 (95% CI: 0.63–0.82).

The receiver operating characteristic (ROC) curve for Transpara AI performance is shown in Figure 7. Transpara AI had fair performance in detecting breast cancer with area under the curve (AUC) 0.73 (95% CI: 0.63–0.82). AI performance stratified by lesion type demonstrated good performance for mass (AUC 0.84, 95% CI: 0.57–1), fair performance for calcifications (AUC 0.70, 95% CI: 0.58–0.82), low performance for asymmetry (AUC 0.69, 95% CI: 0.17–1), and good performance for architectural distortion (AUC 0.81, 95% CI: 0.64–0.98).

## 4. Discussion

This real-world retrospective study evaluated the standalone performance of Transpara AI in detecting breast cancer on screening digital breast tomosynthesis (DBT) exams which proceeded to stereotactic biopsy. Within this high-risk subset, Transpara AI had high sensitivity and high negative predictive value of approximately 95%. The false-positive rate was high with specificity around 25%. Higher AI scores corresponded to increased positive predictive value (PPV). Overall, AI had fair performance in detecting breast cancer with AUC 0.73.

Digital breast tomosynthesis is rapidly emerging as the standard of care in the United States and evaluating AI performance from a standpoint of diagnostic accuracy and workflow efficiency is of utmost importance. Our study provides insights on how to interpret AI risk score in clinical practice and triage screening mammograms. We found low AI risk score (<43) had a malignancy rate of 4.5%, whereas elevated risk (>75) had a malignancy rate of up to 40%. Therefore, we recommend prioritizing elevated-risk exams over low-risk exams to prevent delays in breast cancer diagnosis and treatment. Given the high negative predictive value, AI could potentially be used to decrease the number of unnecessary biopsies. For instance, radiologists could classify a group of punctate calcifications in the setting of low AI risk score as BIRADS-3 (probably benign) instead of BI-RADS 4 (suspicious) with high confidence to avoid biopsy. However, radiologists must exercise caution and use clinical judgment when interpreting AI results to avoid missing breast cancers as our study showed two cases of AI false negatives.

A recent retrospective study of AI-missed cancers on mammograms showed the most common reasons for misses were overlapping dense breast tissue, nonmammary zone locations, architectural distortions, and amorphous microcalcifications [28]. It is imperative for radiologists to be aware of AI shortcomings and pay special attention to certain areas and features. Additionally, AI is prone to generate false positives as evident in our study. Two retrospective studies of DBT screening exams have shown AI false positives are most commonly benign calcifications easily dismissed by radiologists [29,30]. Our dataset was skewed towards calcifications, representing 162 of 212 (76%) stereotactic-biopsy lesions, which may explain the high false-positive rate. Our institution favors ultrasound-guided over stereotactic biopsy due to patient comfort, so masses, asymmetries, and architectural distortions with an ultrasound correlate were less likely to be included. We found AI had fair performance for calcifications with AUC 0.70 (95% CI: 0.58–0.82), indicating scope for improvement with future algorithm development. The expertise of breast radiologists remains valuable and increased knowledge of the reasons for AI misses and false positives may improve patient care.

Our findings showing that AI classified 51% of malignant lesions as elevated risk are in line with a prior retrospective study of 1016 screen-detected cancers in which Transpara 1.7.0 classified 38% of screening mammograms preceding breast cancer diagnosis as high risk [31]. A recent population-based DBT screening cohort study with 4824 exams by Chen et al. using Transpara 1.7.1 reported a sensitivity of 89.2%, specificity of 68.5%, and NPV of 99.8% when defining positive results as intermediate/elevated risk [32]. While our study also showed that AI has a high NPV, we found significantly lower specificity. This may reflect our inherently high-risk subset of patients recommended for stereotactic biopsy as opposed to a general screening population. We also defined a positive AI result as intermediate/elevated risk so specificity could be improved if positive results were defined as elevated risk only.

There has been tremendous growth in the development of deep learning-based AI DBT systems over recent years. As of July 2024, a review article by Lamb et al. states there are at least six FDA-approved applications for screening DBT: Genius AI detection from Hologic Inc. (Marlborough, MA, USA), Lunit INSIGHT DBT from Lunit Inc. (New York, NY, USA), MammoScreen 2D/3D from Therapixel (Nice, France), ProFound AI Software from iCAD Inc. (Nashua, NH, USA), Saige-Dx from DeepHealth Inc. (Somerville, MA, USA), and Transpara from ScreenPoint Medical (Nijmegen, The Netherlands) [33]. While there is limited data comparing the performances of different AI-based systems for DBT, one study showed Profound AI 3.0 outperformed Transpara 1.7.0 (AUC 0.93 and 0.86, respectively; *p* = 0.004) [23]. The rapid deployment of AI systems and constant evolution with subsequent versions poses significant challenges for rigorous validation and reproducibility across institutions and time. We reported AI errors to ScreenPoint Medical for quality assurance and to support further performance improvement in subsequent versions. One false-negative case was identified by the newer version Transpara 2.1.0 which has now become commercially available. A study exploring AI errors found that recent versions of Transpara (1.6.0 and 1.7.0) had lower false-negative rates compared with an earlier version (1.4.0) [34]. There is significant heterogeneity between different AI-based systems for DBT as well as subsequent versions which may impact patient care and outcomes.

There is a gap between AI performance in controlled testing environments and real-life due to complex factors including patient demographics and individual radiologist experience [35]. There are few published studies evaluating AI-based systems for DBT integrated into clinical practice and our study adds to the literature. Important considerations for successful AI implementation include accessibility and equitable performance across diverse patient populations. A recent review article demonstrated that there are limited open access mammography datasets used for development of AI algorithms which report race and ethnicity [36]. If data is missing on certain underserved populations, this may introduce bias and exacerbate health disparities [37,38]. In our small sample of 202 patients, we did not find a statistically significant difference in sensitivity or specificity for AI when stratifying patients by age, race and ethnicity, or breast density. However, further large prospective studies are necessary to ensure AI performs equally well, independent of patient characteristics.

This study had limitations. First, this was an urban multicenter single-institution study focused on a high-risk subset of mammograms which proceeded to stereotactic biopsy, which may limit generalizability. Results may be different for other institutions, including those in other geographic areas with a different patient population. Second, this was a retrospective study limited by a small number of false-negative cancers. Finally, by study design, we focused on lesions prompting stereotactic biopsy, which were predominantly calcifications. The outcomes of artificial intelligence findings not recalled by the radiologist or prompting biopsy through a different modality such as ultrasound or MRI were not evaluated, and this should be explored in future studies.

## 5. Conclusions

DBT is widely adopted in the United States and our study establishes the current value of Transpara AI in detecting breast cancer on screening mammography cases preceding stereotactic biopsy. AI can improve clinical workflow efficiency as a triage tool and can guide radiologists in making follow-up or biopsy recommendations given the high sensitivity and negative predictive value. However, the expertise of radiologists remains valuable as AI is prone to generate false positives and may miss cancers detected by radiologists. Large prospective studies are necessary to validate the performance of AI in the real world and to ensure equitable care across diverse patient populations.

## Figures and Tables

**Table 1:** Patient characteristics. Age is expressed as mean while all other data are expressed as count with percentage in parentheses.

	AI (n = 202)
Mean patient age (y)	57.8
Prior diagnosis of breast cancer, n (%)	24 (12%)
Family history of breast cancer, n (%)	50 (25%)
Breast density, n (%)	
Fatty	11 (5%)
Scattered	87 (43%)
Heterogenous	92 (46%)
Extremely dense	12 (6%)
Race or ethnicity, n (%)	
White	88 (44%)
Black/African American	35 (17%)
Asian	27 (13%)
Hispanic	40 (20%)
Unknown	12 (6%)

**Table 2:** Characteristics of stereotactic biopsy lesions. Data are expressed as count with percentage in parentheses.

	AI (n = 212)
Lesion type	
Mass	10 (5%)
Calcifications	162 (76%)
Asymmetry	15 (7%)
Architectural distortion	25 (12%)
BIRADS score	
0	204 (96%)
1	0 (0%)
2	0 (0%)
3	1 (0.5%)
4	6 (3%)
5	1 (0.5%)
6	0 (0%)
Pathology results:	
Benign	136 (64%)
Atypical with high-risk features *	36 (17%)
Malignant	40 (19%)
DCIS	32
IDC	2
ILC	5
Lymphoma	1

* Atypical with high-risk features: ADH, ALH, LCIS, radial scar/complex sclerosing lesion, and papilloma with ADH.

**Table 3:** Performance of AI in relation to pathology results with contingency table showing true positives (TP), false negatives (FN), false positives (FP), and true negatives (TN).

	Malignant	Benign	
**AI: elevated/intermediate risk**	37 (TP)	130 (FP)	PPV 37/(37 + 130) = 22.2%
**AI: low risk**	2 (FN)	42 (TN)	NPV 42/(42 + 2) = 95.5%
	Sensitivity 37/(37 + 2) = 94.8%	Specificity 42/(42 + 130) = 24.4%	

**Table 4:** Positive predictive value (PPV) of AI by score category.

AI Score	Malignant (n = 39)	Total (n = 211)	% Malignant (PPV)
<40	2	44	4.5%
40–50	5	29	17.2%
50–60	5	41	12.2%
60–70	6	36	16.7%
70–80	7	32	21.9%
80–90	7	22	31.8%
90–100	7	7	100%

## Data Availability

The original contributions presented in the study are included in the article. Further inquiries can be directed to the corresponding authors.

## Abbreviations

AI = artificial intelligence, AUC = area under the ROC curve, CAD = computer-aided detection, DBT = digital breast tomosynthesis, NPV = negative predictive value, PPV = positive predictive value, ROC = receiver operating curve
